# Subacute thyroiditis associated with COVID-19 infection: a report of an increasing entity

**DOI:** 10.20945/2359-3997000000446

**Published:** 2022-01-01

**Authors:** Szofia Hajósi-Kalcakosz, Judit Dénes, Miklós Góth

**Affiliations:** 1 Heim Pál Children's Hospital Department of Infectious Diseases Budapest Hungary Heim Pál Children's Hospital, Department of Infectious Diseases, Budapest, Hungary; 2 Hungarian Defense Forces 2nd Department of Medicine Division of Endocrinology Budapest Hungary Division of Endocrinology, 2nd Department of Medicine, Health Center, Hungarian Defense Forces, Budapest, Hungary

## Abstract

In March 2020, the World Health Organization characterized COVID-19 as a pandemic. By May 2021, 37 cases of subacute thyroiditis (SAT) associated with severe acute respiratory syndrome coronavirus 2 (SARS-CoV-2) had been reported in the literature. We report a patient diagnosed with SAT associated with COVID-19 and review the previously reported cases. A 31-year-old female with no significant previous history developed SAT 5 weeks after SARS-CoV-2 infection. She presented with anterior neck pain and fever. Thyroid function tests revealed hyperthyroidism with slightly increased inflammatory markers. Thyroid ultrasound showed diffuse hypoechoic left lobe and a hypoechoic area in the right lobe. On the fine-needle-aspiration biopsy, large histiocytes, disrupted and normal follicles, and multinucleated giant cells within colloid were seen. Under oral corticosteroid therapy, clinical progression was rapid. Seven weeks later, all thyroid function tests and inflammatory markers normalized. During the recent viral outbreak, clinicians should keep in mind the possibility of SAT after COVID-19, and patients with symptoms of SAT should be tested for SARS-CoV-2.

## INTRODUCTION

In March 2020, the World Health Organization (WHO) characterized COVID-19 as a pandemic. In September 2020, the Endocrine Society published a statement online to alert endocrinologists about the association of SARS-CoV-2 infection and subacute thyroiditis (SAT) based on Brancatella and cols. (1). By May 2021, more than 170 000 000 people were infected with SARS-CoV-2. In addition, 37 cases of SAT, a rare complication of SARS-CoV-2 infection, have been reported in the literature (Table 1) (2-20). On the other hand, it is also important to highlight, as declared in a position statement from the Thyroid Department of the Brazilian Society of Endocrinology and Metabolism, well-controlled hypo- and hyperthyroidism are not associated with an increased risk of COVID-19 infection or severity (21).

**Table 1:** Clinical and biochemical features of the COVID-19 associated SAT cases and the present case

Manuscript	Country	Patient	Time from COVID-19 onset to SAT onset (days)	Laboratory markers at the time of diagnosis	Normal ranges	Initial therapy
Abreu R *et al.* (2)	Brazil	F, 34 y	28	N.A.	N.A.	Prednisone 15 mg/d
Abreu R *et al.* (2)	Brazil	F, 34 y	10	FT4 1.8 ng/dL	N.A.	Prednisone
Abreu R *et al.* (2)	Brazil	F, 39 y	26	N.A.	N.A.	N.A.
Álvarez Martín MC *et al.* (3)	Spain	F, 46 y	Asymptomatic COVID-19, during SAT, SARS-CoV-2 serology positive	TSH 0.11 mIU/mL FT4 2.18 ng/dL ESR 68 mm/h	TSH 0.55-4.78 mIU/mL FT4 0.89-1.76 ng/dL	Prednisone 40 mg/d
Asfuroglu Kalkan E *et al.* (4)	Turkey	F, 41 y	Asymptomatic COVID-19, during SAT, SARS-CoV-2 PCR positive	TSH < 0.008 mIU/L FT4 25.7 pmol/L FT3 7.7 pmol/L CRP 101 mg/L ESR 134 mm/h	TSH < 0.008 mIU/L FT4 12-21 pmol/L FT3 3.1-6.8 pmol/L	Hydroxychloroquine 200 mg/d Prednisone 16 mg/d
Brancatella A *et al.* (1)	Italy	F, 18 y	19	TSH < 0.04 mIU/L FT4 27.2 nmol/L FT3 8.7 pmol/L CRP 6.9 mg/L ESR 90 mm/h	TSH 0.5-4.1 mIU/L FT4 11-23 nmol/L FT3 4.6-8.4 pmol/L	Prednisone 25 mg/d
Brancatella A *et al.* (5)	Italy	F, 38 y	16	TSH 0.1 mIU/mL FT4 29.3 nmol/L FT3 8.0 pmol/L CRP 11.2 mg/L ESR 74 mm/h	TSH 0.4-4.5 mIU/mL FT4 6-16 nmol/L FT3 2.3-4.2 pmol/L	Prednisone 25 mg/d
Brancatella A *et al.* (5)	Italy	F, 29 y	30	TSH < 0.01 mIU/mL FT4 31.8 nmol/L FT3 8.9 pmol/L CRP 7.9 mg/L ESR 110 mm/h	TSH 0.4-4.5 mIU/mL FT4 6-16 nmol/L FT3 2.3-4.2 pmol/L	Prednisone 25 mg/d propranolol 40 mg/d
Brancatella A *et al.* (5)	Italy	F, 29 y	36	N.A.	N.A.	Ibuprofen 600 mg/d
Brancatella A *et al.* (5)	Italy	F, 46 y	20	TSH < 0.01 mIU/mL FT4 27.8 nmol/L FT3 6.9 pmol/L CRP 8 mg/L	TSH 0.4-4.5 mIU/mL FT4 6-16 nmol/L FT3 2.3-4.2 pmol/L	Prednisone 25 mg/d
Campos-Barrera E *et al.* (6)	Mexico	F, 37 y	30	TSH undetectable FT4 1.6 ng/dL CRP 66 mg/L ESR 72 mm/h	N.A.	N.A.
Chakraborty U *et al.* (7)	India	M, 58 y	During SAT, SARS-CoV-2 PCR positive	TSH < 0.005 mIU/L FT4 20.11 ug/dL FT3 2.88 ng/mL CRP 16.6 mg/L ESR 110 mm/h	TSH 0.27-4.2 mIU/L FT4 5.10-14.1 ug/dL FT3 0.80-2.0 ng/mL	Prednisolone 30 mg/d propranolol 40 mg/d
Chong WH *et al.* (8)	USA	M, 37 y	30	TSH 0.01 mIU/L FT4 2.3 ng/dL CRP 14 mg/L ESR 31 mm/h	TSH 0.4-4.5 mIU/L FT4 0.6-1.3 ng/dL	NSAID
Davoodi *et al.* (9)	Iran	M, 33 y	During SAT, SARS-CoV-2 PCR positive	TSH < 0.001 mIU/L tT4 23.1 ug/dL tT3 236 ng/dL CRP 37.9 mg/L ESR 84 mm/h	TSH 0.5-5.0 mIU/L tT4 4-11 ug/dL tT3 75-195 ng/dL CRP <10 mg/L ESR <15 mm/h	Dexamethasone 12 mg/d then prednisone 25 mg/d
Dworakowska *et al.* (10)	UK	F, 57 y	appr. 60	anti-TPO, anti-Tg: positive	N.A.	Ibuprofen, paracetamol
Ippolito S *et al.* (11)	Italy	F, 69 y	5, during SAT, SARS-CoV-2 PCR positive	TSH 0.08 mIU/L FT4 24.6 pg/mL FT3 5.5 pg/mL	TSH 0.27-4.2 mIU/L FT4 0.3-17 pg/mL FT3 2-4.4 pg/mL	Methimazol, methylprednisolone 40 mg/d then prednisone 25 mg/d
Khatri A *et al.* (12)	USA	F, 41 y	28	TSH < 0.008 mIU/L TT4 222.91 nmol/L CRP 36.4 mg/L ESR 107 mm/h	TSH 0.7-4.2 mIU/L TT4 59.34-154.8 nmol/L	Ibuprofen 1,800 mg/d, prednisone 40 mg/d
Mattar SAM *et al.* (13)	Singapore	M, 34 y	9, during SAT, SARS-CoV-2 PCR positive	TSH < 0.01 mIU/L FT4 41.8 pmol/L FT3 13.4 pmol/L CRP 122 mg/L	TSH 0.65-3.70 mIU/L FT4 8.8-14.4 pmol/L FT3 3.2-5.3 pmol/L	Prednisone 20 mg/d, atenolol 25 mg/d
Mehmood MA *et al.* (14)	USA	F, 29 y	49	TSH 0.01 mIU/L FT4 4.4 ng/L FT3 374 ng/L CRP 44 mg/L ESR 84 mm/h	TSH N.A. FT4 0.6-1.3 ng/L FT3 80-150 ng/L	Prednisone 20 mg/d, atenolol 25 mg/d
Muller I *et al.* (15)	Italy	M, 24 y	During SAT, SARS-CoV-2 PCR positive	TSH 0.33 mIU/L FT4 9.6 pmol/L FT3 4.0 pmol/L CRP 10 mg/L	TSH 0.28-4.3 mIU/L FT4 10.3-21.9 pmol/L FT3 3.1-7.7 pmol/L	N.A.
Muller I *et al.* (15)	Italy	F, 59 y	During SAT, SARS-CoV-2 PCR positive	TSH 0.4 mIU/L FT4 16.6 pmol/L FT3 2.3 pmol/L CRP 233 mg/L	TSH 0.28-4.3 mIU/L FT4 10.3-21.9 pmol/L FT3 3.1-7.7 pmol/L	N.A.
Muller I *et al.* (15)	Italy	M, 66 y	During SAT, SARS-CoV-2 PCR positive	TSH 0.43 mIU/L FT4 22.8 pmol/L CRP 52 mg/L	TSH 0.28-4.3 mIU/L FT4 10.3-21.9 pmol/L FT3 3.1-7.7 pmol/L	N.A.
Ruano R *et al.* (16)	Spain	F, 28 y	29	TSH < 0.001 mIU/L FT4 37.5 pmol/L CRP 176 mg/L	TSH 0.38-5.33 mIU/L FT4 7.0-16.0 pmol/L	Paracetamol 1 g/8 h, aspirin 500 mg/d, propranolol 40 mg/d
Ruggeri RM *et al.* (17)	Italy	F, 43 y	cca. 45	TSH 0.006 mIU/L FT4 2.69 ng/dL FT3 7.03 pg/mL	TSH 0.27-4.2 mIU/L FT4 0.7-1.48 ng/dL FT3 1.71-3.71 pg/mL	prednisone 25 mg/d
San Juan MDJ *et al.* (18)	Philippines	F, 47 y	During SAT, SARS-CoV-2 PCR positive	TSH 0.05 mIU/mL FT4 1.68 pg/mL CRP 5.09 mg/dL	TSH 0.47-4.68 mIU/mL FT4 0.78-2.19 pg/mL	NSAID
Seyed Resuli A *et al.* (19)	Turkey	F, 32 y	During SAT, SARS-CoV-2 PCR positive	FT4 26.8 pmol/L FT3 8.4 pmol/L ESR 65 mm/h	FT4 12-22 pmol/L FT3 3-6 pmol/L	Aspirin 3 g/d
Seyed Resuli A *et al.* (19)	Turkey	F, 25 y	During SAT, SARS-CoV-2 PCR positive	FT4 28.1 pmol/L FT3 9.6 pmol/L ESR 58 mm/h	FT4 12-22 pmol/L FT3 3-6 pmol/L	Aspirin 3 g/d
Seyed Resuli A *et al.* (19)	Turkey	F, 45 y	During SAT, SARS-CoV-2 PCR positive	FT4 43.1 pmol/L FT3 14.2 pmol/L ESR 70 mm/h	FT4 12-22 pmol/L FT3 3-6 pmol/L	Aspirin 3 g/d, prednisolone 1 mg/kg/d
Seyed Resuli A *et al.* (19)	Turkey	F, 29 y	During SAT, SARS-CoV-2 PCR positive	FT4 38.1 pmol/L FT3 11.3 pmol/L ESR 65 mm/h	FT4 12-22 pmol/L FT3 3-6 pmol/L	Aspirin 3 g/d
Seyed Resuli A *et al.* (19)	Turkey	F, 21 y	During SAT, SARS-CoV-2 PCR positive	FT4 43.5 pmol/L FT3 16.2 pmol/L ESR 80 mm/h	FT4 12-22 pmol/L FT3 3-6 pmol/L	Aspirin 3 g/d, prednisolone 1 mg/kg/d
Sohrabpour S *et al.* (20)	Iran	F, 26 y	N.A.	TSH 0.07 mIU/L FT4 19.5 pmol/L FT3 18.9 pmol/L CRP 28 mg/L ESR 70 mm/h	TSH 0.4-4.0 mIU/L FT4 12-21 pmol/L FT3 3.1-6.8 pmol/L	Prednisone 25 mg/d
Sohrabpour S *et al.* (20)	Iran	F, 37 y	N.A.	TSH < 0.01 mIU/L FT4 2.3 pmol/L FT3 25.4 pmol/L CRP 38 mg/L ESR 56 mm/h	TSH 0.4-4.0 mIU/L FT4 12-21 pmol/L FT3 3.1-6.8 pmol/L	Prednisone 25 mg/d
Sohrabpour S *et al.* (20)	Iran	M, 35 y	N.A.	TSH 0.12 mIU/L FT4 24.7 pmol/L FT3 19.3 pmol/L CRP 18 mg/L ESR 45 mm/h	TSH 0.4-4.0 mIU/L FT4 12-21 pmol/L FT3 3.1-6.8 pmol/L	Prednisone 25 mg/d
Sohrabpour S *et al.* (20)	Iran	F, 41 y	N.A.	TSH < 0.01 mIU/L FT4 21.9 pmol/L FT3 23.7 pmol/L CRP 43 mg/L ESR 83 mm/h	TSH 0.4-4.0 mIU/L FT4 12-21 pmol/L FT3 3.1-6.8 pmol/L	Prednisone 25 mg/d
Sohrabpour S *et al.* (20)	Iran	M, 52 y	N.A.	TSH 0.17 mIU/L FT4 26.7 pmol/L FT3 21.6 pmol/L CRP 51 mg/L ESR 76 mm/h	TSH 0.4-4.0 mIU/L FT4 12-21 pmol/L FT3 3.1-6.8 pmol/L	Prednisone 25 mg/d
Sohrabpour S *et al.* (20)	Iran	F, 34 y	N.A.	TSH 0.23 mIU/L FT4 18.4 pmol/L FT3 18.1 pmol/L CRP 23 mg/L ESR 39 mm/h	TSH 0.4-4.0 mIU/L FT4 12-21 pmol/L FT3 3.1-6.8 pmol/L	Prednisone 25 mg/d
Hajósi-Kalcakosz Sz *et al.*	Hungary	F, 31 y	35	TSH 0.046 uIU/mL FT4 1.60 ng/dL FT3 4.10 pg/mL CRP 16.9 mg/L ESR 34 mm/h	TSH 0.35-4.94 uIU/mL FT4 0.7-1.48 ng/dL FT3 1.71-3.71 pg/mL	Methylprednisolone 32 mg/d

Abbreviations: appr.: approximately; CRP: C-reactive protein; d: day; ESR: erythrocyte sedimentation rate; F: female, FT3: free triiodothyronine; FT4: free thyroxine; M: male; N.A.: not available; SAT: subacute thyroiditis; TSH: thyroid stimulating hormone; TT4: total thyroxine; y: year.

De Quervain thyroiditis is a known self-limited disease that occurs during or 4-6 weeks after an upper respiratory tract infection caused by several viruses such as influenza, adenovirus, Coxsackie, enterovirus, mumps, measles, Epstein-Barr, and cytomegalovirus, and SARS-CoV-2 appears to be a new emerging causal association (23). The incidence of SAT is 12.1 per 100 000/year, and it is more prevalent in females (19.1 per 100 000/year) than it is in males (4.4 per 100 000/year). The incidence was highest for people 30-40 years old (24 per 100 000/year) and 40-50 years old (35 per 100 000/year), declining with increasing age (23). An association between SAT and HLA-B*35, -B*18:01, -DRB1*01, and -C*04:01 is known, and it might mean that SAT occurs in genetically predisposed people through a susceptibility to viral infections (24-26). It is mainly characterized by a sudden onset of neck pain radiated to the jaw, tenderness, fever, myalgia, and fatigue with the characteristic hyperthyreotic symptoms: palpitation, tremor, diarrhea, and weight-loss (27). The laboratory findings include elevation of erythrocyte sedimentation rate (ESR), C-reactive protein (CRP), high free thyroxine (FT4) and free triiodothyronine (FT3), and suppressed thyroid-stimulating hormone (TSH) in the initial phase without elevation of thyroid autoantibodies (antithyroid peroxidase [TPOAb] and antithyroglobulin [TgAb]). Ultrasound shows normal or slightly enlarged thyroid with focally or diffusely hypoechogenic pattern and low vascularization on color Doppler. Thyroid scintigraphy typically reveals depressed or absent radionuclid uptake. On FNAB cytology multiple multinuclear giant cells with cytoplasm, a dirty background of cellular debris, degenerated-proliferated follicular epithelium cells, rare epithelioid granulomas, and mixed type inflammatory cells are characteristic (28). In some patients, no treatment is required or nonsteroidal anti-inflammatory drugs (NSAIDs) are enough for relief of pain. If the symptoms do not improve, steroid should be administered with daily doses of 20-40 mg prednisone for 2 weeks, which should be tapered down in a period of 6 weeks. In the case of palpitation, beta-blocking agents might be used. Antithyroid drugs that block the synthesis of thyroid hormones are not needed because the excess of thyroid hormones results from the release of preformed thyroid hormones from the inflamed thyroid tissue and not from increased synthesis of thyroid hormones. Subsequently, patients often experience hypothyroidism before returning to euthyroidism. In these cases, transient levothyroxin administration might be needed.

## CASE REPORT

A 31-year-old-woman had symptoms of suspected COVID-19, and on October 2 2020, the reverse transcription-polymerase chain reaction (PCR) for SARS-CoV-2 using nasopharyngeal and oropharyngeal swabs turned out positive. She had 2 days of fever (39.5 °C), severe headache, myalgia, and sleepiness. These symptoms resolved without any medication, and afterwards she had a 10-day-long loss of taste and smell. On October 12, she was completely asymptomatic, but the PCR test remained positive. According to the Hungarian National Guidance, as a health care worker, she went back to work after 3 weeks of disease onset and no further swab tests were performed.

**Figure 1:**
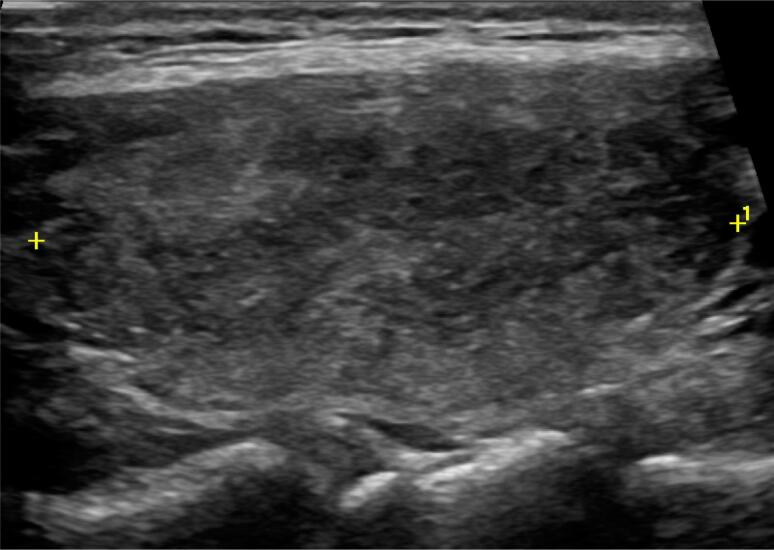
Thyroid ultrasound: diffuse hypoechoic left thyroid lobe.

**Figure 2:**
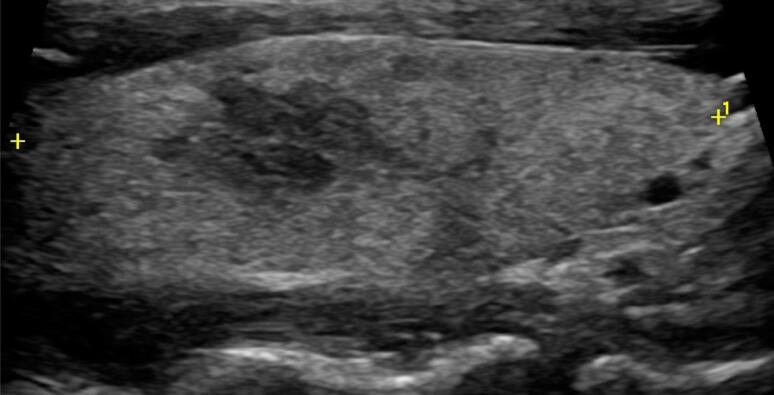
Thyroid ultrasound: in the right thyroid lobe, a 15 × 8 × 7 mm hypoechoic lesion is seen.

On 4 November, after 5 weeks of COVID-19 onset, she developed severe neck pain and tenderness that radiated to the jaw, and a few days later, she experienced fever (38.7 °C), fatigue, myalgia, palpitation, and tremor. Past medical history was unremarkable for thyroid and any other diseases. On 10 November, laboratory test showed high levels of both FT4 1.60 ng/dL (normal range: 0.70-1.48 ng/dL) and FT3 4.10 pg/mL (normal range: 1.71-3.71 pg/mL), low level of TSH 0.046 uIU/mL (normal range: 0.350-4.940 uIU/mL), moderately emerged inflammatory markers (CRP 16.9 mg/L; normal: <10 mg/L), ESR 34 mm/h (normal range: 6-11 mm/h) with normal leukocyte counts and platelets (Table 1). Antibodies (TPOAb and TgAb) were negative. On neck ultrasound, both thyroid lobes had normal size (left lobe 20 × 17 × 39 mm, right lobe 17 × 14 × 43 mm) with normal vascularization. The left lobe was diffuse hypoechoic (Figure 1), and in the right lobe, a 15 × 8 × 7 mm hypoechoic area was detected with slurred border (Figure 2). There was no cervical lymphadenopathy.

**Figure 3:**
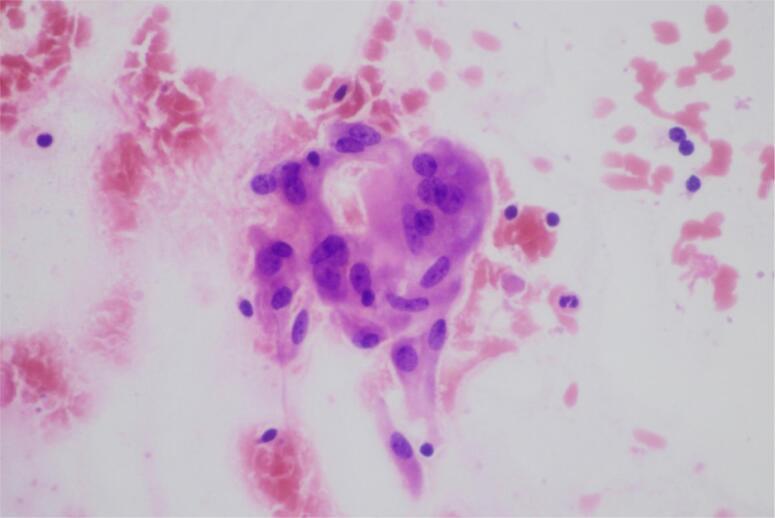
FNAB: small splashes of colloid can be seen with normal-looking follicular cells and 1 multinuclear giant cell.

Because of the lesion in the right lobe, FNAB was performed on 18 November. On the aspiration cytology, large histiocytes, disrupted and normal follicles, and multinucleated giant cells within colloid were seen (Figure 3). The diagnosis of SAT was confirmed. She began NSAID therapy but the symptoms did not improve. She consulted an endocrinologist and began methylprednisolone (32 mg/day for 2 weeks, which was tapered down in 6 weeks). Neck pain and fever disappeared within a day, the other symptoms within 2 weeks. On 24 November, all the inflammatory markers were in a normal range: CRP was 0.1 mg/L and ESR was 7 mm/h. FT4 and FT3 became normal, and TSH was yet suppressed: TSH: 0.019 uIU/mL; FT4: 1.29 ng/dL; and FT3: 3.11 pg/mL. On 26 November, the SARS-CoV-2 IgA, IgM, and IgG were still positive: 18.19 COI. At the last evaluation, on 23 December, she was taking 4 mg/day methyprednisolone and thyroid function tests were in normal range: TSH: 2.558 uIU/mL; FT4: 0.88 ng/dL; and FT3: 2.36 pg/mL.

## DISCUSSION

Although the effect of SARS-CoV-2 virus on the thyroid gland has not yet been fully explored, there are currently four possible hypotheses of the pathogenesis of thyroid dysfunction after COVID-19: (i) direct infection of the thyroid gland by SARS-CoV-2; (ii) underlying nonthyroideal illness syndrome; (iii) dysfunction of the hypothalamic-pituitary-thyroid (HPT) axis causing a diminished level of serum TSH; and (iv) the indirect effects of immune-mediated, postviral inflammatory reaction (29). During the recent outbreak, Muller and cols. (15) reported that angiotensin-converting enzyme 2 (ACE2) receptors, where the SARS-CoV-2 virus binds, are more highly expressed in thyroid cells, than in lung cells. Rotondi and cols. (30). examined 15 patients who underwent thyreoidectomy for benign thyroid nodules and detected ACE2 mRNA in all thyroid samples, which was the first direct proof for the expression of this potential target for SARS-CoV-2 entry.

Interestingly, in patients with severe COVID-19, an increased prevalence of a new phenomenon, thyrotoxicosis secondary to inflammatory or destructive thyroiditis, was observed (31). This is an atypical SAT course noticed especially in hospitalized patients. Lania and cols. (32) retrospectively reported in the THYRCOV study that 20.2% of patients hospitalized for severe COVID-19 presented clinical or subclinical thyrotoxicosis. They suggested that thyrotoxicosis is due to inflammatory or destructive thyroiditis secondary to cytokine storm, because the prevalence of thyrotoxicosis correlated with the concentration of interleukin 6. Muller and cols. (15) also reported increased number of thyrotoxicosis in COVID-19 patients hospitalized in 2020 in high-intensity care units (HICU-20) compared with COVID-19 patients hospitalized in low-intensity care units (LHICU-20) and with patients admitted to the same high-intensity care unit in 2019, before the pandemic (HICU-19). In the HICU-20 group, 15% of patients had thyrotoxicity, and in the HICU-19 and LHICU-20 groups, thyrotoxicity was found in only 1% and 2% of patients, respectively. The patients of the HICU-20 group had significantly lower TSH levels than patients in the LHICU-20 and HICU-19 groups did, and T4 concentrations were significantly higher in the HICU-20 group compared with the LHICU-20 group. In these cases, the lack of neck pain was also noticed. The absence of infiltration due to the SARS-CoV-2-related leukopenia can lead to the lack of tension in the thyroid capsule, which might explain the nonpainful nature of inflammatory or destructive thyroiditis (31).

In the previous coronavirus epidemic that began in 2002, alteration of serum levels of thyroid hormones and TSH was also detected, but there was little evidence of the effects of SARS-CoV-1 on thyroid gland. Only a few histopathological specimens of the thyroid gland were reported. A study by Wei and cols. (33) involving five autopsies of SARS-CoV-1 patients concluded that apoptosis might have played a role in the effect of SARS on thyroid gland. In this context, the increase of proinflammatory cytokines during an acute SARS-CoV-2 infection might be associated with the destructive thyroiditis and thyrotoxicosis.

By May 2021, 37 cases of SARS-CoV-2-associated SAT had been published, and data from these reports are summarized in Table 1 and 2. The past medical history was negative for thyroid diseases in 36 cases; 1 patient had a small, nontoxic, diffuse goiter. It shows that underlying thyroid condition is not a risk factor for SAT. Young and middle-aged female predominance was characteristic as in other cases of SAT. Female-to-male ratio was 3.6:1 (29 females, 8 males), average age 38.5 (range 18 to 69; Table 1). SAT symptoms can copresent with a positive SARS-CoV-2 PCR or even co-occur with the symptoms of COVID-19, which has never been described before in cases of the previously mentioned SAT-causing viruses (3,4,7,9,11,13,15,18,19). The longest period from COVID-19 infection to SAT onset was 49 days. In all of these cases, the clinical course of COVID-19 was asymptomatic or mild, in contrast with destructive thyroiditis. The main presenting symptoms were neck pain radiated to the jaw, thyroid tenderness, and fever, but the number of cases of painless SAT course is increasing. Taking this into account, pain of the thyroid gland is not included in the main diagnostic criteria of SAT reported by Stasiak and Lewiński (25). The characteristic hyperthyreotic symptoms (palpitation, sweating, insomnia, weight-loss, and atrial fibrillation) were also reported. Thyroid function tests were typical: increased thyroid hormones and inflammatory markers as well as decreased TSH were revealed. TSH receptor antibodies were undetectable in all cases. At neck ultrasound, the thyroid was usually enlarged bilaterally with diffuse hypoechoic areas, and low vascularization at color Doppler ultrasonography was characteristic as well. ^99m^Technetium scintiscan showed absent uptake. In most of the cases, steroids and NSAIDs were applied. After beginning of the treatment, symptoms disappeared within a few days. Several weeks after the onset of SAT, all patients’ inflammatory markers were within normal range. In a long-term follow-up, 24 patients were euthyroid, 7 patients were diagnosed with hypothyroidism, and 1 patient still had hyperthyroidism (Table 2).

**Table 2:** Variation of thyroid function in the COVID-19 associated SAT cases described

Manuscript	Patient	Laboratory markers at the time of diagnosis	Thyroid function variation	Normal ranges
Abreu R *et al.* (2)	F, 34 y	N.A.	N.A.	N.A.
Abreu R *et al.* (2)	F, 34 y	FT4 1.8 ng/dL	2.5 weeks later: euthyroid	N.A.
Abreu R *et al.* (2)	F, 39 y	N.A.	N.A.	N.A.
Álvarez Martín MC *et al.* (3)	F, 46 y	TSH 0.11 mIU/mL FT4 2.18 ng/dL ESR 68 mm/h	4 weeks later: hypothyroid TSH 7.75 mIU/mL FT4 0.66 ng/dL	TSH 0.55-4.78 mIU/mL FT4 0.89-1.76 ng/dL
Asfuroglu Kalkan E *et al.* (4)	F, 41 y	TSH < 0.008 mIU/L FT4 25.7 pmol/L FT3 7.7 pmol/L CRP 101 mg/L ESR 134 mm/h	N.A.	TSH < 0.008 mIU/L FT4 12-21 pmol/L FT3 3.1-6.8 pmol/L
Brancatella A *et al.* (1)	F, 18 y	TSH < 0.04 mIU/L FT4 27.2 nmol/L FT3 8.7 pmol/L CRP 6.9 mg/L ESR 90 mm/h	6 weeks later: euthyroid TSH 2.9 mIU/L FT4 16.2 nmol/L FT3 5.3 pmol/L CRP 0.9 mg/L ESR 2 mm/h	TSH 0.5-4.1 mIU/L FT4 11-23 nmol/L FT3 4.6-8.4 pmol/L
Brancatella A *et al.* (5)	F, 38 y	TSH 0.1 mIU/mL FT4 29.3 nmol/L FT3 8.0 pmol/L CRP 11.2 mg/L ESR 74 mm/h	6.5 weeks later: euthyroid	TSH 0.4-4.5 mIU/mL FT4 6-16 nmol/L FT3 2.3-4.2 pmol/L
Brancatella A *et al.* (5)	F, 29 y	TSH < 0.01 mIU/mL FT4 31.8 nmol/L FT3 8.9 pmol/L CRP 7.9 mg/L ESR 110 mm/h	6.5 weeks later: subclinical hypothyroid	TSH 0.4-4.5 mIU/mL FT4 6-16 nmol/L FT3 2.3-4.2 pmol/L
Brancatella A *et al.* (5)	F, 29 y	N.A.	8.5 weeks later: subclinical hypothyroid	N.A.
Brancatella A et al. (5)	F, 46 y	TSH < 0.01 mIU/mL FT4 27.8 nmol/L FT3 6.9 pmol/L CRP 8 mg/L	6.5 weeks later: euthyroid	TSH 0.4-4.5 mIU/mL FT4 6-16 nmol/L FT3 2.3-4.2 pmol/L
Campos-Barrera E *et al.* (6)	F, 37 y	TSH undetectable FT4 1.6 ng/dL CRP 66 mg/L ESR 72 mm/h	4 weeks later: hyperthyroid TSH 0.01 mUI/L	N.A.
Chakraborty U *et al.* (7)	M, 58 y	TSH < 0.005 mIU/L FT4 20.11 ug/dL FT3 2.88 ng/mL CRP 16.6 mg/L ESR 110 mm/h	4 weeks later: hypothyroid TSH 21.29 mIU/L FT4 4.85 ug/dL FT3 0.73 ng/mL	TSH 0.27-4.2 mIU/L FT4 5.10-14.1 ug/dL FT3 0.80-2.0 ng/mL
Chong WH *et al.* (8)	M, 37 y	TSH 0.01 mIU/L FT4 2.3 ng/dL tT3 202 ng/dL CRP 14 mg/L ESR 31 mm/h	3 weeks later: hypothyroid TSH 15 mIU/L FT4 0.1 ng/dL tT3 10 ng/dL	TSH 0.4-4.5 mIU/L FT4 0.6-1.3 ng/dL
Davoodi *et al.* (9)	M, 33 y	TSH < 0.001 mIU/L tT4 23.1 ug/dL tT3 236 ng/dL CRP 37.9 mg/L ESR 84 mm/h	7 weeks later: euthyroid TSH 2.5 mIU/L tT4 6.6 ug/dL tT3 189 ng/dL	TSH 0.5-5.0 mIU/L tT4 4-11 ug/dL tT3 75-195 ng/dL CRP <10 mg/L ESR <15 mm/h
Dworakowska *et al.* (10)	F, 57 y	anti-TPO, anti-Tg: positive	subclinical hypothyroid	N.A.
Ippolito S *et al.* (11)	F, 69 y	TSH 0.08 mIU/L FT4 24.6 pg/mL FT3 5.5 pg/mL	N.A.	TSH 0.27-4.2 mIU/L FT4 0.3-17 pg/mL FT3 2-4.4 pg/mL
Khatri A *et al.* (12)	F, 41 y	TSH < 0.008 mIU/L TT4 222.91 nmol/L CRP 36.4 mg/L ESR 107 mm/h	N.A.	TSH 0.7-4.2 mIU/L TT4 59.34-154.8 nmol/L
Mattar SAM *et al.* (13)	M, 34 y	TSH < 0.01 mIU/L FT4 41.8 pmol/L FT3 13.4 pmol/L CRP 122 mg/L	10 weeks later: euthyroid TSH 2.1 mIU/L FT4 11.0 pmol/L	TSH 0.65-3.70 mIU/L FT4 8.8-14.4 pmol/L FT3 3.2-5.3 pmol/L
Mehmood MA *et al.* (14)	F, 29 y	TSH 0.01 mIU/L FT4 4.4 ng/L FT3 374 ng/L CRP 44 mg/L ESR 84 mm/h	10 weeks later: euthyroid	TSH N.A. FT4 0.6-1.3 ng/L FT3 80-150 ng/L
Muller I et al. (15)	M, 24 y	TSH 0.33 mIU/L FT4 9.6 pmol/L FT3 4.0 pmol/L CRP 10 mg/L	6.5 weeks later: euthyroid	TSH 0.28-4.3 mIU/L FT4 10.3-21.9 pmol/L FT3 3.1-7.7 pmol/L
Muller I *et al.* (15)	F, 59 y	TSH 0.4 mIU/L FT4 16.6 pmol/L FT3 2.3 pmol/L CRP 233 mg/L	8.5 weeks later: euthyroid	TSH 0.28-4.3 mIU/L FT4 10.3-21.9 pmol/L FT3 3.1-7.7 pmol/L
Muller I *et al.* (15)	M, 66 y	TSH 0.43 mIU/L FT4 22.8 pmol/L CRP 52 mg/L	6 weeks later: euthyroid	TSH 0.28-4.3 mIU/L FT4 10.3-21.9 pmol/L FT3 3.1-7.7 pmol/L
Ruano R *et al.* (16)	F, 28 y	TSH < 0.001 mIU/L FT4 37.5 pmol/L CRP 176 mg/L	4.5 weeks later: euthyroid FT4 10.5 pmol/L CRP 2.1 mg/L ESR 56 mm/h	TSH 0.38-5.33 mIU/L FT4 7.0-16.0 pmol/L
Ruggeri RM *et al.* (17)	F, 43 y	TSH 0.006 mIU/L FT4 2.69 ng/dL FT3 7.03 pg/mL	4 weeks later: euthyroid	TSH 0.27-4.2 mIU/L FT4 0.7-1.48 ng/dL FT3 1.71-3.71 pg/mL
San Juan MDJ *et al.* (18)	F, 47 y	TSH 0.05 mIU/mL FT4 1.68 pg/mL CRP 5.09 mg/dL	8 weeks later: hypothyroid TSH 94.3 mIU/mL FT4 0.23 pg/mL FT3 1.20 pg/mL	TSH 0.47-4.68 mIU/mL FT4 0.78-2.19 pg/mL FT3 2.77-5.27 pg/mL
Seyed Resuli A *et al.* (19)	F, 32 y	FT4 26.8 pmol/L FT3 8.4 pmol/L ESR 65 mm/h	2 weeks later: euthyroid	FT4 12-22 pmol/L FT3 3-6 pmol/L
Seyed Resuli A *et al.* (19)	F, 25 y	FT4 28.1 pmol/L FT3 9.6 pmol/L ESR 58 mm/h	3.5 weeks later: euthyroid	FT4 12-22 pmol/L FT3 3-6 pmol/L
Seyed Resuli A *et al.* (19)	F, 45 y	FT4 43.1 pmol/L FT3 14.2 pmol/L ESR 70 mm/h	4 weeks later: euthyroid	FT4 12-22 pmol/L FT3 3-6 pmol/L
Seyed Resuli A *et al.* (19)	F, 29 y	FT4 38.1 pmol/L FT3 11.3 pmol/L ESR 65 mm/h	3 weeks later: euthyroid	FT4 12-22 pmol/L FT3 3-6 pmol/L
Seyed Resuli A *et al.* (19)	F, 21 y	FT4 43.5 pmol/L FT3 16.2 pmol/L ESR 80 mm/h	5 weeks later: euthyroid	FT4 12-22 pmol/L FT3 3-6 pmol/L
Sohrabpour S *et al.* (20)	F, 26 y	TSH 0.07 mIU/L FT4 19.5 pmol/L FT3 18.9 pmol/L CRP 28 mg/L ESR 70 mm/h	4 weeks later: euthyroid TSH 2.21 mIU/L FT4 17.1 pmol/L ESR 17 mm/h	TSH 0.4-4.0 mIU/L FT4 12-21 pmol/L FT3 3.1-6.8 pmol/L
Sohrabpour S *et al.* (20)	F, 37 y	TSH < 0.01 mIU/L FT4 2.3 pmol/L FT3 25.4 pmol/L CRP 38 mg/L ESR 56 mm/h	4 weeks later: euthyroid TSH 1.83 mIU/L FT4 19.3 pmol/L ESR 4 mm/h	TSH 0.4-4.0 mIU/L FT4 12-21 pmol/L FT3 3.1-6.8 pmol/L
Sohrabpour S *et al.* (20)	M, 35 y	TSH 0.12 mIU/L FT4 24.7 pmol/L FT3 19.3 pmol/L CRP 18 mg/L ESR 45 mm/h	4 weeks later: euthyroid TSH 3.75 mIU/L FT4 13.2 pmol/L ESR 9 mm/h	TSH 0.4-4.0 mIU/L FT4 12-21 pmol/L FT3 3.1-6.8 pmol/L
Sohrabpour S *et al.* (20)	F, 41 y	TSH < 0.01 mIU/L FT4 21.9 pmol/L FT3 23.7 pmol/L CRP 43 mg/L ESR 83 mm/h	4 weeks later: euthyroid TSH 1.84 mIU/L FT4 18.9 pmol/L ESR 25 mm/h	TSH 0.4-4.0 mIU/L FT4 12-21 pmol/L FT3 3.1-6.8 pmol/L
Sohrabpour S *et al.* (20)	M, 52 y	TSH 0.17 mIU/L FT4 26.7 pmol/L FT3 21.6 pmol/L CRP 51 mg/L ESR 76 mm/h	4 weeks later: euthyroid TSH 0.46 mIU/L FT4 20.1 pmol/L ESR 28 mm/h	TSH 0.4-4.0 mIU/L FT4 12-21 pmol/L FT3 3.1-6.8 pmol/L
Sohrabpour S *et al.* (20)	F, 34 y	TSH 0.23 mIU/L FT4 18.4 pmol/L FT3 18.1 pmol/L CRP 23 mg/L ESR 39 mm/h	4 weeks later: euthyroid TSH 3.67 mIU/L FT4 12.7 pmol/L ESR 2 mm/h	TSH 0.4-4.0 mIU/L FT4 12-21 pmol/L FT3 3.1-6.8 pmol/L
Hajósi-Kalcakosz S *et al.*	F, 31 y	TSH 0.046 uIU/mL FT4 1.60 ng/dL FT3 4.10 pg/mL CRP 16.9 mg/L ESR 34 mm/h	7 weeks later: euthyroid TSH: 2.558 uIU/mL FT4: 0.88 ng/dL FT3: 2.36 pg/mL	TSH 0.35-4.94 uIU/mL FT4 0.7-1.48 ng/dL FT3 1.71-3.71 pg/mL

Abbreviations: F: female; FT3: free triiodothyronine; FT4: free thyroxine; M: male; N.A.: not available; SAT: subacute thyroiditis; TSH: thyroid stimulating hormone; TT4: total thyroxine; TT3: total triiodothyronine; y: year.

In our case, the age and the gender were typical for SAT. The patient presented with SAT symptoms after 5 weeks of COVID disease onset, which is also an average time. Clinically, neck pain and fever were the main presenting symptoms. In the acute phase, the characteristic laboratory markers were observed: a marked elevation of inflammatory markers and primary hyperthyroidism. At neck ultrasound, the thyroid size and the vascularization were normal, which is uncommon in SAT. One possible explanation is that ultrasonography was performed relatively early after the symptom onset. Due to the hypoechoic area with slurred border detected in the right lobe, an urgent FNAB was advised to rule out malignancy. To date, this is the fourth case of SARS-CoV-2-associated SAT where FNAB was taken. In our case, large histiocytes, disrupted and normal follicles, and multinucleated giant cells within colloid were observed (Figure 3). Abreu and cols. (2) reported 3 cases of SAT where FNAB was taken. All 3 cytological pictures were very similar; they all showed clusters of epitheloid histiocytes forming loose granulomas and scattered giant cells amid rare inflammatory and follicular cells, similar to our specimen. Because of the definitive diagnosis of SAT according to the FNAB findings, radionuclide thyroid scanning was not performed on our patient. Under oral corticosteroid therapy, a rapid clinical progression was observed. In a long-term follow-up, after 7 weeks of SAT onset, the patient was euthyroid, as were the majority of the previously reported cases.

During the currently ongoing pandemic, SARS-CoV-2 must be thought of as an etiological agent of De Quervain thyroiditis, and thyroid citology would be advisable for the better understanding of the effects of SARS-CoV-2 on thyroid gland.

In summary, we presented a case report of SAT associated with SARS-CoV-2 and the fourth case in which FNAB was performed. In this article, we also summarized the previously reported SAT cases associated with SARS-CoV-2.

In conclusion, we would like to emphasize that during the recent viral outbreak, clinicians should keep in mind the possibility of SAT after COVID-19. According to the high prevalence of asymptomatic COVID-19 cases, during the ongoing COVID-19 pandemic, patients presenting with SAT should be tested for SARS-CoV-2 serology or PCR. We would like to advise further research to understand the possible genetic predilections and histopathological changes of SARS-CoV-2 associated with SAT.
